# 220 A Comparative Analysis of Academic and Policymaker Priorities in Physical Activity Policy and Its Infrastructure Support

**DOI:** 10.1093/eurpub/ckae114.033

**Published:** 2024-09-26

**Authors:** Kevin Volf, Oisin Connaughton, Catherine Woods

**Affiliations:** University of Limerick, Limerick, Ireland; University of Limerick, Limerick, Ireland; University of Limerick, Limerick, Ireland

## Abstract

**Purpose:**

Physical activity (PA) researchers regularly seek to impact public policy. However, policymakers may perceive the relevance of policy evidence differently to researchers, thus causing a barrier to research utilization. The Physical Activity Environment Policy Index (PA-EPI) is a 45-indicator policy assessment tool, designed to be used in collaboration with policymakers, to assess both PA policies themselves and also the infrastructure that supports their implementation. This study aimed to identify which of the PA-EPI indicators were rated most important for increasing population levels of PA, and if there was consensus or differences in opinion between academics and policymakers.

**Methods:**

This study is a secondary analysis on data collected from academic experts and policymakers as part of the development of the PA-EPI. Policymakers (n = 40) and academics (n = 50) were asked to rate the importance of each of the 45 PA-EPI indicators on a 10-point Likert scale (1= not at all important to 10 = extremely important for increasing population levels of PA). Differences between the mean importance ratings of policymakers and academics were tested using the Mann Whitney U test.

**Results:**

Mean importance ratings of policymakers and academics were strongly correlated (r = 0.82, p < 0.001 for PA policies themselves and r = 0.77, p = < .001 for policy infrastructure support). Academics rated every indicator higher compared to policymakers, except for the two indicators of the Mass Media subdomain. The indicator receiving the highest level of importance for both policymakers and academics was in the transport domain (Indicator T01). Three indicators were rated significantly different in the domains of Urban Design (indicator UD03; p = 0.027), Funding and Resources (FR03; p = 0.014) and Health in all Policies (HIAP02; p = 0.039).

**Conclusion:**

The findings suggest that policymakers and academics have similar perceptions of the importance of a range of different physical activity policy indicators. These findings present a challenge to theories of research utilization which propose that policymakers and academics are separate, isolated groups with differing views of the evidence supporting different policy propositions.

